# The Effect of Chronic Ozone Exposure on the Activation of Endoplasmic Reticulum Stress and Apoptosis in Rat Hippocampus

**DOI:** 10.3389/fnagi.2016.00245

**Published:** 2016-10-25

**Authors:** Erika Rodríguez-Martínez, Concepcion Nava-Ruiz, Elsa Escamilla-Chimal, Gabino Borgonio-Perez, Selva Rivas-Arancibia

**Affiliations:** ^1^Departamento de Fisiologia, Facultad de Medicina, Universidad Nacional Autónoma de México (UNAM)Ciudad de Mexico, Mexico; ^2^Laboratorio de Neuropatologia Experimental, Instituto Nacional de Neurologia y Neurocirugia Manuel Velasco SuarezMexico City, Mexico; ^3^Facultad de Ciencias, Departamento de Ecologia y Recursos Naturales, Universidad Nacional Autónoma de México (UNAM)Ciudad de Mexico, Mexico

**Keywords:** ozone, oxidative stress, endoplasmic reticulum stress, apoptosis

## Abstract

The chronic exposure to low doses of ozone, like in environmental pollution, leads to a state of oxidative stress, which has been proposed to contribute to neurodegenerative disorders, including Alzheimer’s disease (AD). It induces an increase of calcium in the endoplasmic reticulum (ER), which produces ER stress. On the other hand, different studies show that, in diseases such as Alzheimer’s, there exist disturbances in protein folding where ER plays an important role. The objective of this study was to evaluate the state of chronic oxidative stress on ER stress and its relationship with apoptotic death in the hippocampus of rats exposed to low doses of ozone. We used 108 male Wistar rats randomly divided into five groups. The groups received one of the following treatments: (1) Control (air); (2) Ozone (O_3_) 7 days; (3) O_3_ 15 days; (4) O_3_ 30 days; (5) O_3_ 60 days; and (6) O_3_ 90 days. Two hours after each treatment, the animals were sacrificed and the hippocampus was extracted. Afterwards, the tissue was processed for western blot and immunohistochemistry using the following antibodies: ATF6, 78 kDa glucose-regulated protein (GRP78) and caspase 12. It was also subjected to terminal deoxynucleotidyl transferase dUTP nick end labeling (TUNEL) assay and electronic microscopy. Our results show an increase in ATF6, GRP78 and caspase 12 as well as ER ultrastructural alterations and an increase of TUNEL positive cells after 60 and 90 days of exposure to ozone. With the obtained results, we can conclude that oxidative stress induced by chronic exposure to low doses of ozone leads to ER stress. ER stress activates ATF6 inducing the increase of GRP78 in the cytoplasm, which leads to the increase in the nuclear translocation of ATF6. Finally, the translocation creates a vicious cycle that, together with the activation of the cascade for apoptotic cell death, contributes to the maintenance of ER stress. These events potentially contribute in the neurodegeneration processes in diseases like AD.

## Introduction

Environmental pollution has become a world public health issue while ozone (O_3_) is one of the main photochemical air pollutants. The chronic exposure to low doses of O_3_ causes a state of oxidative stress in the brain of rats. Additionally, it produces alterations in the endogenous antioxidant systems as well as an increase in the levels of proteins and oxidized lipids (Rivas-Arancibia et al., 2010; Rodríguez-Martínez et al., 2013; Gómez-Crisóstomo et al., 2014; Poljšak and Fink, 2014).

Furthermore, lipid oxidation and cell membrane proteins cause disturbances in the permeability of the membrane, leading to an increase of the ions within the cell. Calcium is one of the most abundant ions in the organisms and extracellular liquid. Intracellular calcium is finely regulated since it activates a number of signaling pathways including apoptotic cell death pathways. During the chronic state of oxidative stress there is a calcium entry into the cell and into the endoplasmic reticulum (ER), one of the organelles in charge of maintaining the intracellular calcium homeostasis along with the mitochondrion.

On the other hand, the ER is involved in a number of cell functions such as: quality control of protein synthesis, post-translational modification and protein folding. These processes are regulated by different enzymes (Paschen and Doutheil, 1999; Ellgaard and Helenius, 2003; Anelli and Sitia, 2008), also known as molecular chaperones, are proteins in the ER that respond to different calcium concentrations and are involved in new protein folding (Corbett and Michalak, 2000; Molinari and Helenius, 2000). For instance, the binding immunoglobulin protein (BiP)/78-kDa glucose-regulated protein (GRP78), an ER chaperone regulating those pathways, is bound to three transmembrane ER proteins: ATF6, inositol-requiring enzyme 1a (IRE1a) and protein kinase RNA-like endoplasmic reticulum kinase (PERK; Kincaid and Cooper, 2007). The binding of BiP/GRP78 to such transmembrane proteins takes place in the ER as a response to calcium stress (Li et al., 1993). Furthermore, these transmembrane proteins regulate cell death along with the massive calcium entry through both the activating factors at transcriptional level (CHOP or XBP1) and the caspases activation in response to an extended ER stress (Lin et al., 2007; White-Gilbertson et al., 2013). During ER stress, BiP/GRP78 triggers a series of events that lead to the nuclear translocation of ATF6 (Haze et al., 1999). ATF6 is a type II transmembrane protein with a C-terminal located in the ER lumen and an N-terminal in the cytosol (Ye et al., 2000).

If the ER eventually shows chronic stress and does not manage to recover homeostasis, it triggers cell apoptosis via caspases activation. Among these caspases, caspase 12 is one of the enzymes involved in the cell death cascade. Caspase 12 is located in the outer ER membrane activated by ER stress (Nakagawa et al., 2000; Yoneda et al., 2001) and is expressed in cortical and hippocampal neurons (Shimoke et al., 2011; Dlugos, 2014). Caspase 12 activates caspase 3 (Mehmet, 2000; Hitomi et al., 2004), leading to apoptotic death. The objective of this study was to evaluate the effect of chronic oxidative stress on ER stress and its relationship with apoptotic death in the hippocampus of rats repeatedly exposed to low doses of ozone.

## Materials and Methods

Animal care and handling were in accordance with the Norma Official Mexicana NOM-036-SSA2-2002 and approved by the Institutional Committee for the Care and the Use of Laboratory Animals (CICUAL), of the Medicine School at the Universidad Nacional Autónoma de México.

One hundred eight Wistar male rats weighing 250–300 g were individually housed in acrylic boxes with free access to water and food (Purina, Minnetonka, MN, USA) and were kept in a room with clean air. They were randomly divided into six experimental groups (*n* = 18). Each group received one of the following treatments: Control (exposed to ozone-free air), 7, 15, 30, 60 and 90 days of ozone exposure (daily exposure to 0.25 ppm of ozone for 4 h; Rivas-Arancibia et al., 2010). Immediately after the ozone exposure was completed, animals were returned to their home cages. Two hours after the last air or ozone exposure, all the animals were deeply anesthetized using 50 mg/kg of sodium pentobarbital, and put to death. The brain was removed and the hippocampus of each animal was dissected, isolated and analyzed using one of the following techniques: (1) Western blot for evaluation of ATF6, GRP/78 and caspase 12 (*n* = 6); (2) Terminal deoxynucleotidyl transferase dUTP nick end labeling (TUNEL) staining (*n* = 6) and immunohistochemistry for ATF6, GRP/78 and caspase 12; and (3) Electron microscopy (*n* = 6).

### Ozone Exposure

Animals were placed inside a chamber with an air diffuser connected to a variable-flux ozone generator (5 L/s). The same chamber was used to treat the control group with an airflow free of ozone and the groups exposed to ozone. Animal ozone exposure has been previously described by Pereyra-Muñoz et al. (2006) and Rivas-Arancibia et al. (2010).

### Determination of Protein

Hippocampal samples were treated with 0.017% deoxycholate and precipitated with 6% trichloroacetic acid (Bensadoun and Weinstein, 1976). After centrifugation at 5000 g for 30 min at 4°C, the protein content was determined as described by Bradford (1976). BSA was used as a standard.

### Electrophoretic Techniques and Western Blot Analysis

The hippocampus of each of the groups exposed to ozone were homogenized, and SDS-PAGE was performed according to the Laemmli method. The proteins were separated in a 10% polyacrylamide gel under denaturing conditions. After the run, the proteins were electrotransferred to a polyvinylidene difluoride membrane (Immobilon P; Millipore) in a semidry electroblotting system (Bio-Rad) at 25 V for 50 min. Membranes were blocked by incubation in a buffer containing 20 mM Tris base, pH 7.5, 500 mM NaCl, 0.05% Tween-20 (TBS-T buffer) and 5% blotting grade nonfat-dry milk (BioRad). Then, they were tested with ATF6 (1:500, ABCAM ab11909), GRP/78 (1:900, ABCAM ab21685), anti-caspase 12 (1:500, ABCAM ab62484) or GAPDH (1:10,000, Cell Signaling #2118). The membranes were incubated for 2 h with the secondary antibody at an adequate dilution in the TBS-T buffer. After three washes with TBS-T buffer, bands were visualized using horseradish peroxidase-conjugated goat anti-rabbit IgG (Pierce) at a dilution of 1:10,000 and using the Enhanced ChemiLuminescence assay (Amersham Life Science) according to the manufacturer’s instructions. ATF6, GRP/78 and caspase 12 were normalized using anti-GAPDH as loading control to confirm equal amounts of protein.

### Immunohistochemistry

Brain sagittal sections containing the hippocampus were deparaffinized. Afterwards, the tissue was hydrated; to this end, sections of different groups were placed on a hydration train in the following order: xilox, absolute alcohol, 96°, 70° alcohol, 50° alcohol and distilled water during 2 min for each of the previous steps. The tissue was placed into a heat retrieval solution (Biocare Medical, Concord, CA, USA) and into an electric pressure cooker (Decloacking Chamber, Biocare Medical) for 20 min. After washing with distilled water and treating with hydrogen peroxide (diluted 1:5; Fisher Scientific, Santa Fe Springs, CA, USA) for 10 min, sections were rinsed again with distilled water and treated with a blocking reagent (Background Sniper, 4 plus Detection Component, Biocare Medical) for 1 h. They were then washed with 0.1 M phosphate–buffered saline (PBS), pH 7.4 (Merck, Darmstadt, Germany) and incubated for 12 h at 4°C with anti-ATF6 (mouse monoclonal antibody, diluted 1:200, ABCAM ab11909), anti-GRP78 (rabbit polyclonal antibody, diluted 1:200, ABCAM ab21685) and anti-caspase 12 (rabbit polyclonal antibody, diluted 1:200, ABCAM ab62484). The sections were rinsed with PBS and treated with biotinylated secondary antibody (Universal Link, Biocare Medical) for 2 h. After washing with PBS, they were treated with streptavidin-enzyme conjugates (4 plus detection component, streptavidin-hrp, Biocare Medical) for 2 h, and washed again with PBS. The bound antibody was visualized using 3,3′-diaminobenzidine (DAB Substrate Kit, ScyTek, West Logan, UT, USA) as the chromogen; the same length of exposure to DAB was used for all samples. The slices were washed in distilled water and counterstained with hematoxylin-buffer solution. Slices were covered with Permount, while the sections of dentate gyrus of hippocampus were examined with a BX41 Olympus Microscope and photographed using an Evolution-QImagin Digital Camera Kit (MediaCybernetics, Silver Spring, MD, USA).

### TUNEL

Sagittal brain sections embedded in paraffin were deparaffinized, and then treated to TUNEL assay, following supplier instructions (Apoptosis detection kit, TA300, R&D Systems, Minneapolis, MN, USA). To finish, tissue was rinsed, dried and coverslipped with Permount (Fisher Scientific, Pittsburgh, PA, USA). Tissue slices were used to detect DNA fragmentation by TUNEL assay.

### Electron Microscopy

For the ultrastructural study, the animals were anesthetized with pentobarbital and perfused through the heart with fixative solution (2.5% glutaraldehyde in 0.1 M Sorensen’s phosphate buffer solution, pH 7.3). The brain tissue was postfixed with 1% osmium tetroxide and embedded in epoxy resin. Semi-thin sections were stained with toluidine blue and examined with a light microscope. Ultrathin sections were then contrasted with uranyl acetate and lead citrate and were examined using a Carl Zeiss EM10 transmission electron microscope. The criteria to assess harm in the ER included edema and discontinuity in the ER.

### Statistics

The data obtained were analyzed through the Kruskal-Wallis and Mann-Whitney U nonparametric tests. The values of *P* < 0.05 were considered statistically substantial.

## Results

### Effect of Oxidative Stress on ATF6 Expression in Hippocampus of Rats Exposed to Ozone

**Figure 1:**
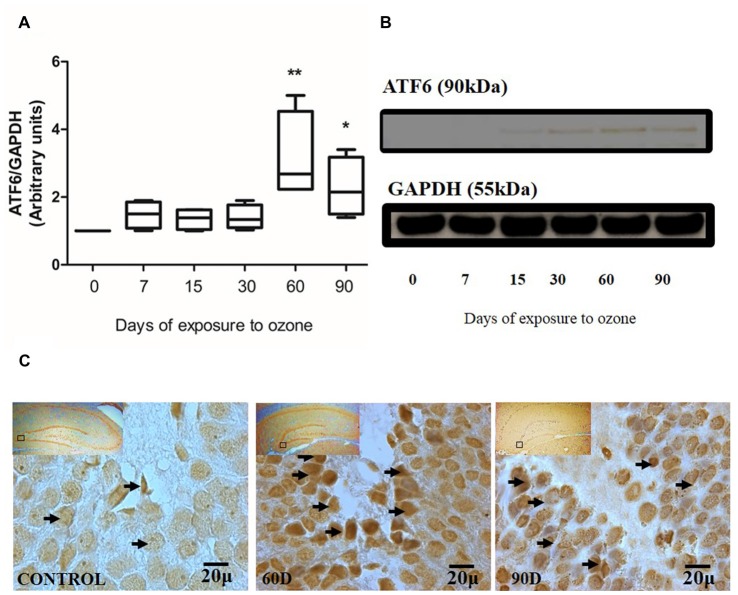
**Effect of chronic exposure to ozone on ATF6 expression in rat hippocampus. (A)** Shows the densitometry analysis of the protein levels for ATF6 from rat hippocampus following exposure to ozone for 7, 15, 30, 60 and 90 days. Note the significant increase in the expression of ATF6 at 60 and 90 days of treatment. **(B)** The results show an increase in the protein expression following 60 (**p < 0.05) and 90 days of exposure to ozone (*p < 0.05) compared with the control groups. GAPDH staining was used as an internal control. **(C)** Effects of ozone treatments on ATF6 immunoreactivity in the dentate gyrus in rat hippocampus. Light photomicrographs show immunoreactivity of ATF6 in cells from hippocampus of rats exposed to ozone (arrows). The control group showed low immunoreactivity. We observed ATF6 translocation to the nucleus of the cells and an increase of immunoreactivity for ATF6 at 60 and 90 days of exposure to ozone. Arrows show ATF6 immunoreactivity 100×. The square indicates the area that is shown as 100× magnification.

The chronic exposure to low doses of ozone causes changes in ATF6 expression in the hippocampus, depending on the time of exposure. The results of the western blot densitometric analysis for ATF6 show an important increase of ATF6 in the group of 60 and 90 days of exposure to ozone with respect to the control group (*P* < 0.05; Figures 1A,B). Additionally, the micrographs of the ATF6 immunohistochemistry in the dentate gyrus of rats exposed to ozone show an increase in immunoreactivity and a higher nuclear translocation of ATF6 at 60 and 90 days of exposure to ozone with respect to the control group (Figure 1C).

### Effect of Oxidative Stress on GRP78 Expression in Hippocampus of Rats Exposed to Ozone

**Figure 2:**
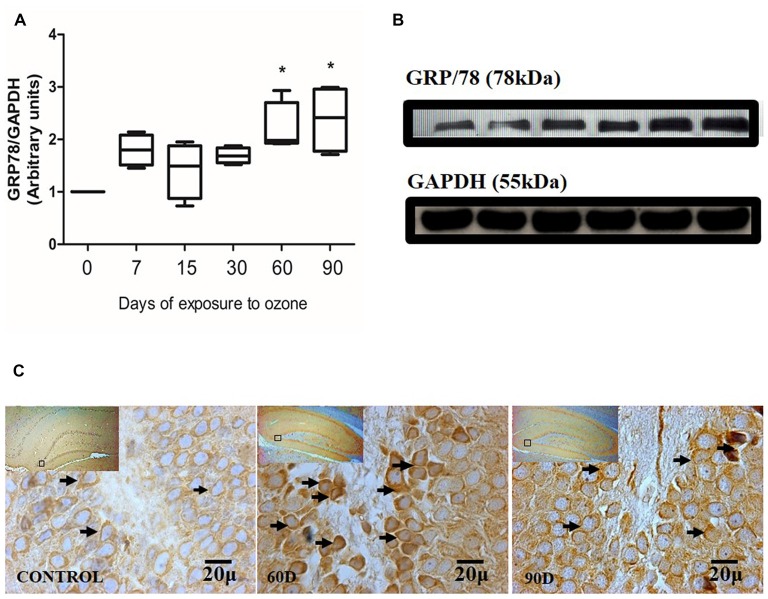
**Effect of chronic exposure to ozone on 78-kDa glucose-regulated protein (GRP78) expression in rat hippocampus. (A)** Shows the densitometry analysis of the protein levels for GRP78 from rat hippocampus following exposure to ozone for 7, 15, 30, 60 and 90 days. There is a significant increase at 60 and 90 days of treatment. **(B)** The results show an increase in the protein expression following 60 and 90-day exposure to ozone (*p < 0.05) compared with the control groups. GAPDH staining was used as an internal control. **(C)** Effects of ozone treatments on GRP78 immunoreactivity in the dentate gyrus of rat hippocampus. Light photomicrographs show immunoreactivity in GRP78 of cells from hippocampus of rats exposed to ozone. The control group showed low immunoreactivity. We observed an increase of immunoreactivity for GRP78 at 60 and 90 days of exposure to ozone. Arrows show GRP78 immunoreactivity 100×. The square indicates the area that is shown as 100× magnification.

Chronic oxidative stress produced by low doses of ozone produces alterations in the expression of GRP78 depending on the time of exposure. The western blot densitometric analysis for GRP78 shows a tendency towards increase from 7, 15 and 30 days of exposure to O_3_. However, at 60 and 90 days of exposure to ozone, our results show a significant increase with respect to the control group (*P* < 0.05; Figures 2A,B). The micrographs of the dentate gyrus immunohistochemistry of rats exposed to ozone for GRP/78 show an increase in the immunoreactivity at 60 and 90 days of exposure to ozone with respect to the control group (Figure 2C).

### Effect of Chronic Exposure to Ozone in Caspase-12 Expression in Hippocampus of Rats Exposed to Ozone

**Figure 3:**
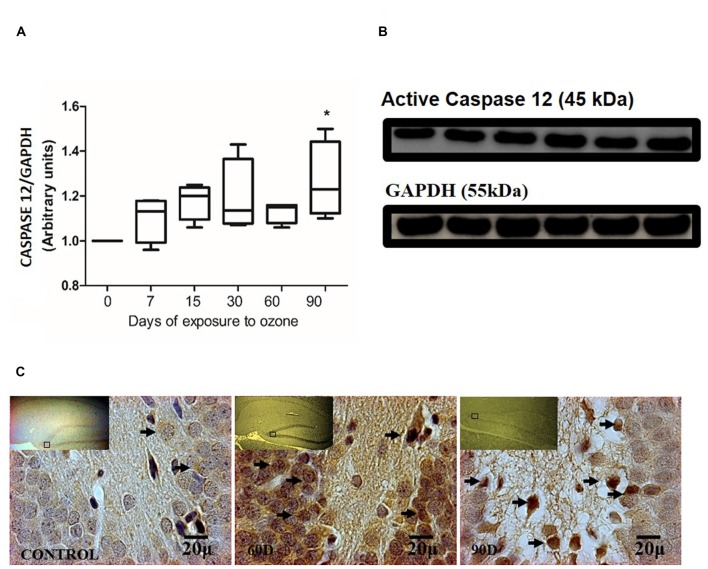
**Effect of chronic exposure to ozone on caspase 12 expression in rat hippocampus. (A)** Shows the densitometry analysis of the protein levels for caspase 12 from rat hippocampus following exposure to ozone for 7, 15, 30, 60 and 90 days. There is a significant increase at 90 days of exposure to ozone. **(B)** The results show an increase in the protein expression following 60 and 90-day exposure to ozone (*p < 0.05) compared with the control groups. GAPDH staining was used as an internal control. **(C)** Effects of ozone treatments on caspase 12 immunoreactivity in the dentate gyrus of rat hippocampus. Light photomicrographs show immunoreactivity for caspase 12 in cells from hippocampus of rats exposed to ozone. The control group showed low immunoreactivity. We observed an increase of immunoreactivity for caspase 12 at 60 and 90 days of exposure to ozone. Arrows show caspase 12 immunoreactivity 100×. The square indicates the area that is shown as 100× magnification.

The chronic exposure to ozone induces a state of oxidative stress together with an increase in the expression of caspase-12. The analysis of caspase-12 shows an increase between 7 and 60 days of exposure to ozone. However, the results of the statistical analysis demonstrate a significant increase of the protein at 90 days of exposure to ozone with respect to the control group (*P* < 0.05; Figures 3A,B). The micrographs of the dentate gyrus immunohistochemistry of rats exposed to ozone for caspase 12, show an increase in the immunoreactivity at 60 and 90 days of exposure to ozone with respect to the control group (Figure 3C).

### Effect of Chronic Exposure to Ozone in TUNEL in Hippocampus of Rats Exposed to Ozone

**Figure 4:**
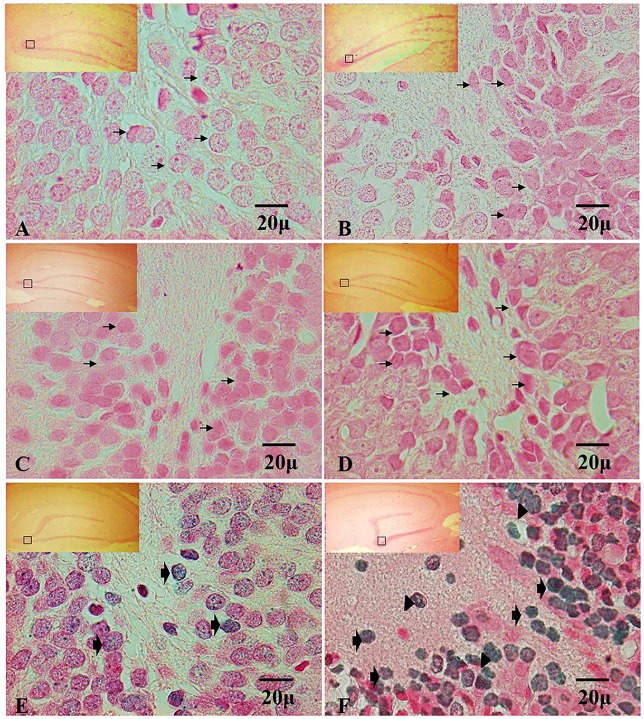
**Effect of chronic exposure to ozone on terminal deoxynucleotidyl transferase dUTP nick end labeling (TUNEL) positive cells in rat hippocampus.** Micrographs of dentate gyrus of hippocampus of rats exposed to ozone. **(A)** Control, **(B)** 7 days (D), **(C)** 15D, **(D)** 30D, **(E)** 60D and **(F)** 90D. Micrographs **(A–D)** show non-positive cells for TUNEL (arrows show healthy cells). Micrographs **(E,F)** show an increase in TUNEL-labeled cells which displayed densely labeled, with different types of chromatin condensation. We observe chromatin grouped around the margin of the nucleus (broad arrow), chromatin condensation located in the center of the nucleus (arrowhead) and several after 60 and 90 days of exposure to ozone 100×. The square indicates the area that is shown as 100× magnification.

The analysis of the TUNEL positive cells have not shown immunoreactivity in the dentate gyrus in hippocampi exposed to air (control group), in the control groups, nor at 7, 15 and 30 days of exposure to ozone (Figures 4A–D). In contrast, at 60 and 90 days of exposure to ozone, a sharp increase in the immunoreactivity is observed with respect to the control group. We also detected different types of nuclear chromatin condensation at 60 and 90 days of exposure to ozone with respect to the control group (Figures 4E,F).

### Effect of Oxidative Stress on the Ultrastructural ER Changes of Hippocampi of Rats Exposed to Ozone

**Figure 5:**
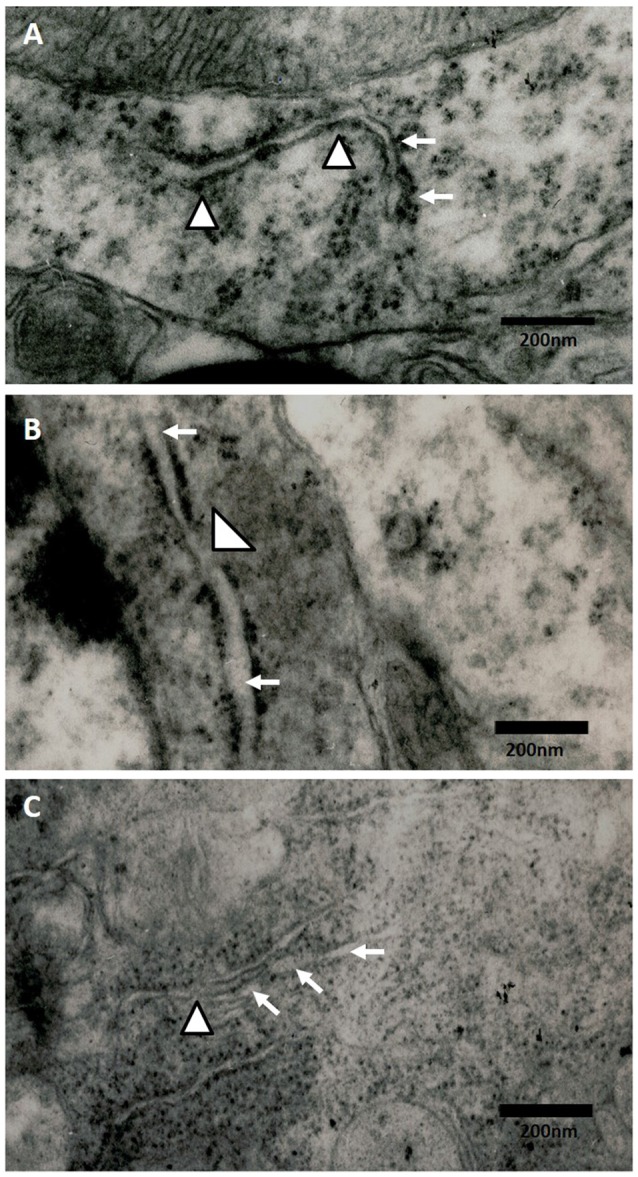
**Effect of chronic exposure to ozone on endoplasmic reticulum (ER) in rat hippocampus.** Electron micrographs of rat hippocampus exposed to ozone. **(A)** Control, **(B)** 60D, **(C)** 90D. **(A)** The micrograph shows the ER of normal appearance with associated ribosomes. **(B,C)** show damage and swelling of ER (broad arrow) and loss of continuity in the membranes of ER (arrowhead) after 60 days and 90 days of exposure to ozone 40,000×.

The electronic microscopy micrographs of the hippocampus of rats exposed to air (control group) show that the ER is conserved. We also observed membrane-bound ribosomes in the conserved ER of the control group (Figure 5A). At 60 days of exposure to ozone, the electronic micrographs of rat hippocampus show a swollen ER and the loss of membrane-bound ribosomes with respect to the control group (Figure 5B). At 90 days of exposure to ozone, the electronic micrographs of hippocampus show damage to the ER and membrane discontinuity. A decrease in the quantity of membrane-bound ribosomes was observed with respect to the control group (Figure 5C).

## Discussion

The repeated exposure to low doses of ozone produces a state of chronic oxidative stress, causing damage and cell death (Rivas-Arancibia et al., 2010). The increase of oxidative stress is accompanied by calcium entry into the cell. In response to the cell stress caused by changes in the calcium concentration either exposure to free radicals or glucose decrease, ER stress is produced, leading to alterations in functions and build-up of misfolded proteins in the ER (Ozcan and Tabas, 2012). In consequence, signaling pathways known as response to misfolded proteins are activated. This response has been interpreted as a compensation mechanism to correct the alteration (Paschen and Doutheil, 1999). However, when ER stress is maintained, a greater quantity of reactive oxygen species (ROS) is generated. This has an impact in the control of the levels of misfolded proteins in the ER, leading to damage in the cell functions (Chao et al., 2013).

Our results show a significant increase of both ATF6 (Figure 1) and GRP78 (Figure 2) at 60 and 90 days of exposure to ozone. It is known that the response control to misfolded proteins in ER stress is mainly regulated by GRP78 and ATF6 (Kincaid and Cooper, 2007). This shows that oxidative stress produced by ozone induces an increase of GRP78. The increase may activate the nuclear translocation of ATF6 in response to misfolded proteins as observed in Figure 1. Additionally, GRP78 dissociation promotes ATF6 activation and the subsequent activation of genes with the capability to re-establish folding during ER stress (Fonseca et al., 2013). In our results we see a non-significant increase in the expression of GRP78 and ATF6 from 7 to 30 days of exposure to ozone. During these periods of time, the cells are still able to compensate the damage produced by chronic exposure to low doses of ozone. However, despite the significant increase in GRP78 and ATF6, the system is unable to reverse or compensate the damage produced at 60 and 90 days of exposure, leading to cell death from apoptosis, induced by changes in calcium in the ER; the changes result in the activation of caspase 12. This shows that the increase in caspase-12 expression at 90 days of exposure to ozone (Figure 3) is related to the increase in the number of TUNEL positive cells (Figure 4) where it is evident that the system cannot fight the damage produced by chronic exposure to low doses of ozone. On the other hand, the ozone not only induces changes at a molecular lever but also ER ultrastructural alterations. Our results show ER membrane dilation as well as a decrease in the membrane-bound ribosomes from 60 to 90 days of exposure to the gas (Figures 5B,C). The morphological alterations are the final result of important failures in the molecular, biochemical and physiological mechanisms of the cell (Chang, 1993). In contrast, the loss of calcium homeostasis is the most immediate and adverse effect caused by oxidative stress induced by exposure to ozone.

The results indicate that ER stress may be playing an important role in the apoptotic damage induced by chronic exposure to low doses of ozone, as shown in Figure 3 where it is seen that ER stress induces caspase-12 release.

Based on these data, we propose that ozone causes ER stress, which induces an increase of caspase 12 and neuronal cell death. Both the oxidative stress and ER damage have been associated to a wide number of degenerative diseases including Alzheimer’s disease (AD). It is known that environmental pollution directly impacts health; however, there are few studies dedicated to clarifying the role ozone plays in the neurodegeneration processes and its possible participation in neurodegenerative diseases like Alzheimer’s. This study demonstrates that the exposure to low doses of ozone has a deleterious effect by inducing a state of oxidative stress which, in turn, leads to the activation of apoptotic death mediated by ER stress. This may contribute to the maintenance and progression of the chronic process of neurodegeneration in Alzheimer’s patients.

## Author Contributions

ER-M was involved in the immunohistochemistry, western blot, TUNEL, electron microscospy techniques and in writing the article draft. CN-R was involved in carrying out the electron microscopy. EE-C was involved in the TUNEL assay. GB-P carried out the exposure of animals to ozone, taking samples for different techniques. SR-A supervised the project, was involved in the design and coordination and revised the article draft.

## Funding

This work was supported by the Dirección General de Apoyo al Personal Académico (Grant no. IN221114 to SR-A).

## Conflict of Interest Statement

The authors declare that the research was conducted in the absence of any commercial or financial relationships that could be construed as a potential conflict of interest.
